# Prediction of mono- and di-nucleotide-specific DNA-binding sites in proteins using neural networks

**DOI:** 10.1186/1472-6807-9-30

**Published:** 2009-05-13

**Authors:** Munazah Andrabi, Kenji Mizuguchi, Akinori Sarai, Shandar Ahmad

**Affiliations:** 1 National Institute of Biomedical Innovation, 7-6-8 Saito-Asagi, Ibaraki-shi, Osaka, Japan; 2 Graduate School of Frontier Biosciences, Osaka University, Osaka, Japan; 3 Department of Bioscience and Bioinformatics, Kyushu Institute of Technology, Fukuoka, Japan

## Abstract

**Background:**

DNA recognition by proteins is one of the most important processes in living systems. Therefore, understanding the recognition process in general, and identifying mutual recognition sites in proteins and DNA in particular, carries great significance. The sequence and structural dependence of DNA-binding sites in proteins has led to the development of successful machine learning methods for their prediction. However, all existing machine learning methods predict DNA-binding sites, irrespective of their target sequence and hence, none of them is helpful in identifying specific protein-DNA contacts. In this work, we formulate the problem of predicting specific DNA-binding sites in terms of contacts between the residue environments of proteins and the identity of a mononucleotide or a dinucleotide step in DNA. The aim of this work is to take a protein sequence or structural features as inputs and predict for each amino acid residue if it binds to DNA at locations identified by one of the four possible mononucleotides or one of the 10 unique dinucleotide steps. Contact predictions are made at various levels of resolution viz. in terms of side chain, backbone and major or minor groove atoms of DNA.

**Results:**

Significant differences in residue preferences for specific contacts are observed, which combined with other features, lead to promising levels of prediction. In general, PSSM-based predictions, supported by secondary structure and solvent accessibility, achieve a good predictability of ~70–80%, measured by the area under the curve (AUC) of ROC graphs. The major and minor groove contact predictions stood out in terms of their poor predictability from sequences or PSSM, which was very strongly (>20 percentage points) compensated by the addition of secondary structure and solvent accessibility information, revealing a predominant role of local protein structure in the major/minor groove DNA-recognition. Following a detailed analysis of results, a web server to predict mononucleotide and dinucleotide-step contacts using PSSM was developed and made available at  or .

**Conclusion:**

Most residue-nucleotide contacts can be predicted with high accuracy using only sequence and evolutionary information. Major and minor groove contacts, however, depend profoundly on the local structure. Overall, this study takes us a step closer to the ultimate goal of predicting mutual recognition sites in protein and DNA sequences.

## Background

Protein-DNA interactions have been the subject of extensive investigation in recent years [1-8]. Some of these studies have focussed on predicting transcription factor binding sites on DNA [9-11], whereas others focus on the prediction of a novel protein to be potentially DNA-binding [12-14]. Earlier, we have analyzed the sequence and structural features of DNA-binding sites in proteins and developed methods for their prediction using neural networks [15,16]. Similar and more accurate methods have since been reported [17-20].

Although these methods have been successful in quickly identifying DNA-binding residues, they all fall short of predicting specific protein-DNA interactions. So far, the prediction of specificity has been achieved by using computationally demanding methods, such as knowledge-based potentials derived from the structures of protein-DNA complexes or the docking of modelled DNA structures to unbound protein structures [21,22]. There are also studies exploiting comparative modelling techniques as well as knowledge compiled from mononucleotide contacts [23,24]. Despite their success in understanding protein-DNA interactions, limitations in their application persist. The docking approaches require full-length target DNA response elements and solved protein structures, whereas comparative modelling requires at least one and perhaps several homologous proteins to be solved in complex with DNA. Application of the knowledge-based approaches has also been limited to relatively low resolution mononucleotides, whereas many DNA structural properties leading to recognition by proteins actually depend on at least more detailed context such as dinucleotide sub-sequence.

In the current study, we make a novel attempt to apply neural network based methods to predict whether a residue binds to a specific mono- or di-nucleotide DNA subsequence. To achieve this goal, we employ three different schemes for defining a specific environment. In the first scheme, we perform statistical analysis of contacts between each of the 20 amino acids with each of the four nucleic acid types (Ade, Cyt, Gua and Thy; called mononucleotide in this work) and attempt to predict such contacts for each residue in a protein chain using neural networks. In the second scheme, we distinguish between the side chain/main chain of an amino acid residue and the side chain (nucleotide base)/backbone of each of the four nucleic acid types, giving rise to a total of 16 contact types, followed by their prediction. Finally in the third scheme, the analysis and prediction of contacts is carried out at a dinucleotide level, i.e., a pair of successive nucleotides in a DNA sequence (with 10 unique combinations from a total of 16 possibilities). Rigorous neural network models with strict cross-validation are employed for prediction, where the neural network input is the feature information from the protein residues and the target output is a multidimensional vector whose dimension is defined by the number of independent DNA-contacts possible in a given specificity definition. The prediction performance is evaluated for various input feature sets and specificity definitions, as well as data subsets divided in terms of secondary structure and solvent accessibility.

## Methods

**Figure 1:**
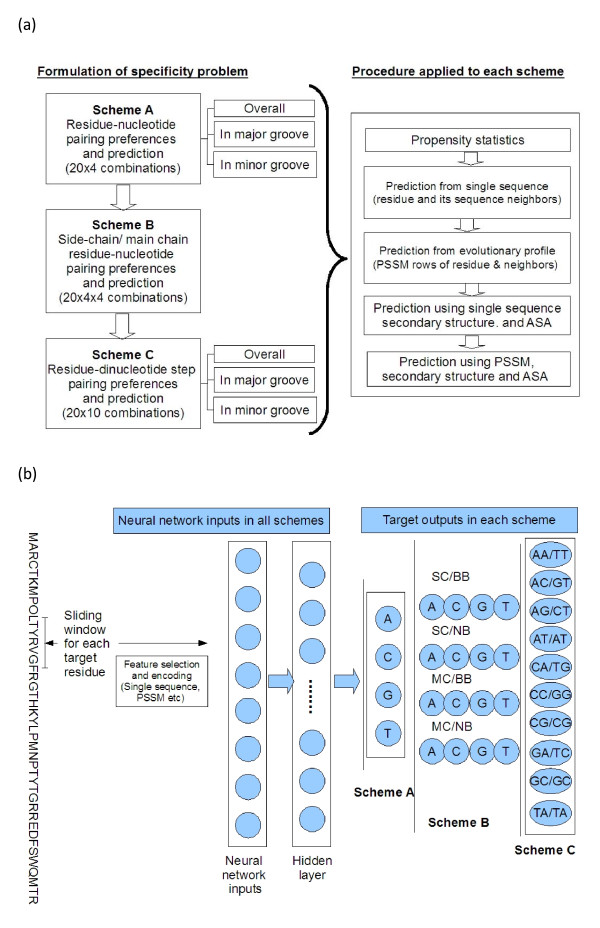
**(a) Overall layout of the current study and (b) prediction workflow in each scheme**. SC and MC stand for protein side chain and main chain atoms and BB and NB stand for DNA backbone and nucleic acid base respectively.

Figure 1 illustrates the methods adopted in a nutshell. Three schemes are employed to define the types of contacts observed between protein and DNA. A common procedure for analysis and prediction is, then, applied to each scheme.

### Contact definition schemes

In the following, a contact is defined if the distance between any atoms in the protein and DNA entities is less than 3.5Å, and the atom selection satisfies the condition of a given contact definition scheme. The major and minor groove atoms are defined by assuming a canonical B-DNA structure [25].

#### Scheme A (mono-nucleotide contacts)

In this scheme a specific contact of each amino acid residue is classified into four mononucleotide types (Ade, Cyt, Gua or Thy). Scheme A is implemented in three ways; overall (including all contacts), major groove (including contacts only in the DNA major groove), and minor groove (including contacts only in the DNA minor groove). Thus, three sets of 80 propensity values each are analyzed. In terms of prediction, it implies that for each amino acid residue for each implementation, a four-dimensional binary vector has to be predicted, whose bit values correspond to contacts with a mononucleotide type.

#### Scheme B (side-chain/main-chain contacts)

Both protein and DNA are made of continuous sequence of atoms directly connected to atoms from the next residue or nucleotide (they are sugar-phosphate atoms together called backbone in DNA, and N, C, C_α _and O atoms called main chain in protein). These backbone or main chain atoms are common to all residue and nucleotide types and therefore as such look like being non-specific. However, even the backbone atoms carry indirect information about the side chain (protein) or nucleic acid base (DNA) to which they are attached, primarily due to the structural constraints. In case of DNA, this effect is often measured in terms of "indirect readout" mechanism, based on sequence-dependent conformation [21]. It is therefore interesting to look at the contacts formed by these pairs of atomic groups from protein and DNA. Scheme B, tries to do exactly the same.

In this scheme 16 types of protein-DNA contacts are defined for each amino acid residue. They consist of four types of contacts for each of the four mononucleotides; between (i) protein main chain and DNA backbone (MC-BB), (ii) protein main chain and DNA nucleic acid base (MC-NB), (iii) protein side chain and DNA backbone (SC-BB) and (iv) protein side chain and DNA nucleic acid base (SC-NB). This gives 20 × 16 propensity values for the analysis, and a 16-dimensional binary vector for prediction.

#### Scheme C (dinucleotide step contacts)

There are several studies, which deal with DNA-recognition in terms of direct and indirect mechanisms, latter of which relies on sequence dependent conformational energy of DNA [26]. Conformational energy of DNA has usually been characterized by stacking deformations in adjacent pair of nucleotides (together with their complementary dinucleotide on the other strand), called base-steps [27]. Thus, in Scheme C, we tried to look at the prediction of protein-DNA contacts from the perspective of dinucleotide steps.

In this scheme, a specific contact is defined in terms of the identity of two consecutive nucleotides in DNA. In double stranded DNA (considered for this study), complementary base pairing occurs between dinucleotides; for example, an Ade-Cyt (AC) dinucleotide is complemented by Thy-Gua (written in the reverse order as GT). Therefore, dinucleotide sequence elements – also called dinucleotide steps – are written along with their complementary dinucleotide steps (e.g., GT/AC represent Gua-Thy from one strand and Ade-Cyt from the other). Since the two strands are equivalent, the AC/GT and GT/AC steps are redundant and there are only 10 unique dinucleotide steps, viz. AA/TT, AC/GT, AG/CT, AT/AT, CA/TG, CC/GG, CG/CG, GA/TC, GC/GC and TA/TA, respectively [27].

In terms of the propensity statistics, this scheme leads to 20 × 10 = 200 possible scores and in terms of the prediction, a 10-dimensional binary vector, in which each bit represents the contact state of a residue with a specific dinucleotide step.

Similar to scheme A, the dinucleotide step contacts are further divided into the major and minor groove contacts for a more detailed analysis.

### Data set (PDNA159) and contact calculation

A complete list of protein-DNA complexes, with a resolution better than 2.5 Å and solved by X-ray crystallography, was downloaded from the Protein Data Bank (PDB) [28] on August 29, 2007. A total of 670 protein-DNA complexes were obtained, consisting of 1178 protein chains. Protein chains with less than 40 residues were removed, leaving 1171 chains. Data redundancy was removed by clustering the protein sequences using BLASTCLUST [29] at a 25% sequence identity threshold. At this threshold 208 clusters were obtained, from which protein chains interacting with single stranded DNA were removed. From each of the remaining 192 clusters, the protein chain with the highest number of DNA contacts was selected. The protein structures were then manually examined and in a few cases the protein chain with the highest contacts was replaced by another member, if the former had some obvious structural defects such as a substantial number of missing atoms or chain breaks in the protein and/or DNA. A finally selected list of 159 protein chains, along with their structural class (using a known scheme [30]) is provided in Additional file 1: Table S1.

### Contact statistics and propensity values

We used two measures to evaluate significantly overpopulated contacts in the data. First, propensity scores were calculated for each contact type (see below). Second, we computed the expected number of contacts in each category using random docking (see below) and made a comparison with the observed number of contacts. For our discussions in the results sections, we primarily depend on the propensity scores, as the numerical values provided are more intuitive and easier to compare between two different types of contacts.

#### Propensity scores

Propensity score is defined for each of the 20 amino acid residues having a specific type of contact in each scheme. For example, the propensity of Arg contacting with Ade (in scheme A) is obtained by first calculating the relative frequency of contacts (defined by the number of Arg-Ade contacts divided by the number of Arg residues in the database) and then normalizing it by the relative frequency of contacts with Ade made by all amino acid residues. More formally, the propensity *P*_*ij *_of amino acid type *i*, to have a contact of type *j *(defined in a given contact definition scheme) is given by:



where ρ_*ij *_is the number of contacts between amino acid type *i *and contact type *j *in a given scheme relative to the total number of amino acid residues of type *i *in the data, and ρ_*j *_is the number of amino acid residues of any type having contact type *j *relative to the total number of amino acid residues in the data.

#### Statistical significance

Propensity scores described above can be computed either for each protein or by pooling the contact counts from multiple proteins. Propensity scores derived from a single protein are unreliable because of the small counts, whereas by pooling the entire data, we cannot directly estimate the statistical significance of the difference between two sets of scores. To deal with this problem, we created an ensemble of random protein lists, of size equal to that of the overall data. Each random list of proteins is created by successively selecting one protein from the overall data after replacement of the drawn sample. In this way, an ensemble of 50 random lists of 159 proteins each were created from PDNA159, allowing a protein to be selected more than once or not at all in the process of randomization. This sampling method allows for calculating the stable values of standard deviations in propensity scores. The statistical significance is calculated by comparing differences between the mean propensities obtained from these distributions. Student's t-test was used to determine whether the difference between a pair of score is statistically significant. This test was conducted using the *t_test_2 *module of the octave open source programming environment . A non-parametric test using *Mann-Whitney-Wilcoxon's u-test *was also carried out and gave similar results and hence the results from that test are not reported.

#### Random Docking and expected number of contacts

To perform statistical tests based on the counts from the entire dataset, we need to calculate the expected number of contacts. This number cannot be obtained directly from the overall statistics of the pooled counts because each complex has a different nucleotide and amino acid composition and the contacts are further constrained by the structural features of each complex. A random docking procedure has been developed earlier to calculate the expected number of contacts between amino acids and nucleic acid bases [31]. According to this procedure, the expected number of contacts between a mononucleotide and an amino acid residue corresponds to the product of the effective number of mononucleotides and the effective number of amino acids in a protein-DNA complex. The effective number of mononucleotides and amino acids is computed by summing up their relative accessible surface areas. A total number of expected counts is obtained by adding the protein-wise values. We extended this procedure to all contact types considered in this work. This required a rigorous calculation of accessible surface areas of all atomic groups and summing up only the subset of atoms considered in the definition of contact type. The expected number of contacts is provided in the supplementary data [see Additional file 2] of statistics together with the observed counts in each category of contacts.

In the results section, the *p*-values obtained from the *t*- or *chi-square *tests have not been discussed explicitly but these values are reported in the supplementary data [see Additional file 2]. Whenever a comparison is discussed in results, the p-value from the *t-test *is less than 0.05, implying that the difference has at least 95% significance.

### Prediction method

All predictions are made using neural networks, to approximate a function relating the residue-wise environments of a protein to the contact space defined by a contact definition scheme. Further explanation for the neural network architecture, training and performance evaluation is provided in the following.

#### Neural network inputs

#### Sparse encoding of a residue's sequence environment

Two types of predictions have been attempted in general. In the first case, single protein sequences are used to derive information of a residue and its immediate neighbours with a varying window size. In the second case each residue is represented by its evolutionary profile derived from a multiple alignment with similar proteins (see the next subsection). The first type of information (an amino acid residue and its sequence neighbours) is encoded by 21-bit sparse-encoded binary vectors such that in each block of 21 units, all units except for the one identifying a given residue are set to zero. The first 20 units represent an amino acid type and the last is used to label terminal positions, where no neighbour is present. This type of encoding was first used by Qian and Sejnowski [32] for secondary structure prediction and has been employed in several more studies since then.

#### Evolutionary profiles (PSSM)

Position specific scoring matrices (PSSMs) representing the evolutionary profile of a residue is generated by using the PSI BLAST[29] program and searching NCBI's NR database for each protein sequence, in much the same way as previous work by us and others [16,17]. Three iterations of PSI BLAST were used to generate PSSMs with default parameters of *blastpgp*. The first 20 columns of PSSM rows are used for neural network input, identifying the log-odds values of evolutionary occurrences of 20 types of residues in given sequence positions. Since these values have a wide range, they are transformed by a *sigmoidal *function such that all PSSM inputs to the neural network range between 0 and 1.

#### Local structure and global amino acid information

Single sequence information encoded by sparse vectors or multiple alignment information consisting of rows of PSSM is also enriched by attaching structural information on the target residues in terms of their accessible surface areas (ASA) and secondary structure (SS). In addition, to each input pattern the global amino acid composition (GAC) is also added to formulate a more detailed description of the residue environment. The ASA and SS were calculated using the DSSP program [33] and the GAC is simply the 20-dimensional relative amino acid composition within the protein. A three-bit vector is used to represent the secondary structure in which the first, second and third bits encode the presence of helix, strand or "any other" secondary structure.

The target (or desired output) vectors for neural networks are (i) 4 or 16-bit vectors representing contacts and contact types in the case of mononucleotide and (ii) 10-bit vectors representing a dinucleotide step to which a residue is in contact.

#### Design, training and validation

All neural networks used in this work are three layered (one hidden layer) fully connected neural networks, simulated using SNNS software [34] and trained using the standard back propagation method. Typical number of hidden units was twice the number of dimensions in the target vector. Window sizes based on 1 to 8 sequence neighbours were tested and the best performing combination for a given scheme was retained. Throughout the study we used a five-fold cross-validation scheme. The list of 159 proteins was divided into five parts and for each training cycle, three sets were combined to form the training data. Out of the two subsets left out, the first is used to determine the stopping point of training and the second is left out of the process of training. After the training is completed, the performance on the left-out data set is evaluated. All performance scores reported are computed on these left out sets. Five cycles of cross-validation, by shuffling the training and test data sets ensure that each protein has been used for evaluating performance.

### Performance evaluation

Trained neural networks return a numerical value between 0 and 1 for each residue, which may be transformed to a binary state of binding or non-binding by choosing a cut-off.

For each contact type, the existence of a contact is considered positive and negative otherwise. True means that the predicted and observed contact states are identical and false implying otherwise. The TP, FP, TN, FN values correspond to a given cut-off, at which neural network analogue values are transformed into binary predictions.



#### Area under the curve (AUC of ROC)

Sensitivity and specificity are computed over the entire range of predicted real values by using different cut-offs to transform them into binary predictions. ROC graphs are then plotted by showing "1-specificity" (false positive rate) on the x-axis and sensitivity (true positive rate) on the y-axis. This graph shows how the false positive rate increases with an increase in the true positive rate. The total area under this curve (AUC) is computed, which is used as a measure of performance throughout this work. This provided a single performance measure for each prediction. When comparing the prediction performance for any pair of contact types, five values are used to determine the standard deviations (or error bars).

## Results and discussion

### Mononucleotide recognition

#### Overall contact statistics

Propensity scores of all amino acid residue types for each of the four mononucleotides, viz Ade, Cyt, Gua and Thy, in the 159 protein chains are analyzed [see Additional file 1: Table S7]. The results are in broad agreement with the preferences of base-amino acid contacts reported in other similar studies, although there are some differences in data selection, redundancy removal and scoring procedures [31,35-37].

#### Overall prediction

**Figure 2:**
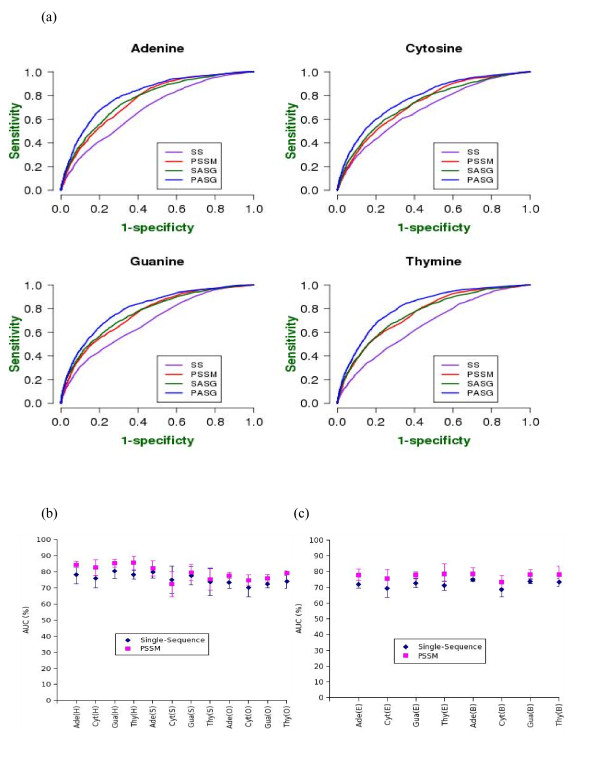
**Prediction performance of mononucleotide-specific contacts using various descriptors**. (a) Overall performance (b) performance classified by secondary structure (c) performance classified by solvent accessibility. All performance estimates are in the percentage units of area under the ROC curve (AUC). **Abbreviation**: SS: Single sequence information. PSSM: prediction using PSSM, SASG: prediction using single sequence, solvent accessibility and secondary structure of target residue, and global amino acid composition of the protein chain. PASG: prediction using PSSM, solvent accessibility and secondary structure of target residue, and global amino acid composition of the protein chain.

Figure 2a shows the results of mononucleotide specific residue contacts using all four variants of prediction [numerical values in Additional file 1: Table S9]. The results indicate that single sequences, i.e., a residue and its sequence neighbours can correctly classify residues as binding to individual mononucleotides with 66–68% accuracy (measured by the AUC of the ROC curve; see Methods). These scores are significantly improved, if evolutionary information (PSSM) is used instead of single sequence neighbours (AUC ranging between ~73–76%). Both these scores are comparable to the prediction performance reported earlier for the sequence-based prediction of DNA-binding sites without identifying a mononucleotide type, which shows that the (local) amino acid sequence and evolutionary information carry a significant part of the information required to predict not only the DNA-binding sites in general, but also the nucleotide to which they are likely to bind. About 7–8 percentage point improvement in prediction is introduced by PSSM over single sequence. This shows that the evolutionary patterns of amino acid substitutions in given positions are significantly constrained by the requirements for specific and non-specific recognition of DNA by proteins. When the structural information of the residues and the overall amino acid composition of the protein are added to the inputs, the single sequence and PSSM-based predictions are improved by ~7 and ~4 percentage points, respectively. The smaller improvement in the PSSM-based predictions is apparently due to the fact that the residue substitution patterns encoded in the PSSM implicitly contain some information about the structure. Indeed, the predictions from single sequence plus structural information are almost as accurate as PSSM alone, supporting the argument of structural information being present in the PSSM. Among the four mononucleotide types, the prediction performance does not vary significantly, with the differences typically less than 2 percentage points.

We examined whether the prediction performance by using structural information is significantly biased in favour of any secondary structure type or between buried and exposed regions. This is achieved by separating the predicted results in terms of their secondary structure or solvent accessibility. Figures 2b and 2c depict the prediction performances within each structural class defined by secondary structure or solvent accessibility. Most performance differences were shown to be statistically significant between helical, strand and other secondary structure positions, as well as between buried and exposed regions. We observe that both helical and strand positions are better predicted than the other secondary structures. Using more accurate PSSM based predictions; helical residues were much better predicted than any other structures. The most significant difference comes for Cyt contacts, for which helical residue binding is predicted by about 10 percentage points more accurately then strand, which in turn is about 4 percentage points better than other structures. Differences in prediction performance for buried and exposed residues were relatively smaller, with PSSM predictions showing almost no difference. This is true for all but Cyt-contacting residues, where buried positions are better predicted than the exposed ones in all cases.

#### Major and minor groove contact statistics

##### Major and minor groove contacts are NOT exclusive

It is believed that proteins recognize DNA and bind to either its major groove or minor groove and based on this DNA-binding proteins are often categorised as those binding to the major or minor groove. This classification may suggest that the major and minor groove contacts are exclusive within a given complex. However, we observe that only a small number of proteins form contacts exclusively in the major groove (46 Proteins) or the minor groove (16 Proteins) in our dataset, whereas most other proteins, especially those with larger contacting interface form contacts in both major and minor grooves. Also, the actual number of contacts in either groove is much smaller than the contacts with the DNA backbone [see Additional file 1: Table S2, S3 and S4], reiterating the fact that most of the protein-DNA complex stability should come from non-groove contacts [31]. Groove contacts are believed to contribute significantly to specific recognition processes (in accordance with the classic work of Seeman et al. [38]). This stronger role of groove contacts in specificity is also indicated by their strong structure dependence (see next subsections on prediction).

##### Mononucleotide-amino acid pair propensities

**Figure 3:**
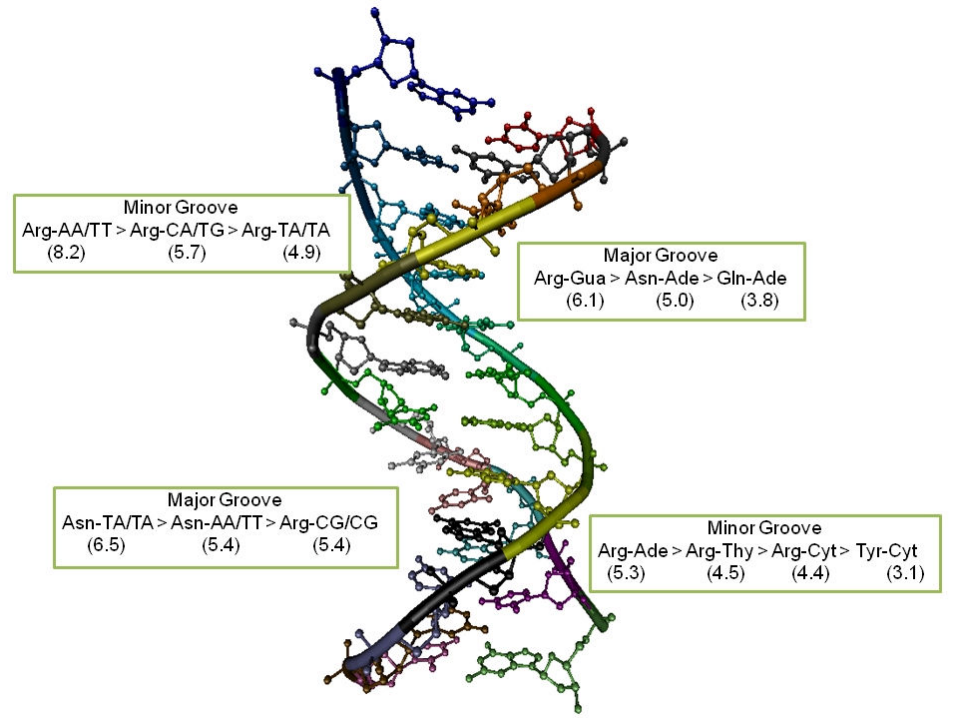
**Highest propensity residue-mononucleotide (right) and residue-dinucleotide step (left) pairs in major and minor DNA-grooves**.

Mononucleotide-amino acid propensities in the major and minor grooves are shown in Additional file 1: Table S8. Comparison between propensities is illustrated in Figure 3. Key observations are enumerated as follows:

Propensity differences between residues in the major groove are much higher than those in the minor groove:

The minor groove propensity scores show that except for Arg and to some extent Tyr, most other residues have similar affinity for all mononucleotides (values close to 1). However, in the major groove many residues (for example, Asn preferring Ade with propensity ~5.0, His preferring Gua with propensity ~3.0, both Asp and Glu preferring Cyt with propensity ~2.0) show a greater variation. The lower residue-wise specificity in the minor groove might be an indication of the essential role of structure in protein-DNA recognition in the minor groove. This subsequently implies a greater role of indirect recognition through the DNA-backbone in these interactions.

High Arg-Gua propensities in major groove are replaced by Arg-Ade pairs in the minor groove:

Whereas Arg-Gua pair does have a very high propensity (>6.0) in the major groove as expected, Arg seems to prefer Ade in the minor groove (propensity>5.0). The propensity of Arg for Ade in the major groove and Gua in the minor groove is lower in comparison (both less than 3.0). This is a significant observation as Arg-Gua has been widely known as a preferred residue-base pair in all protein-DNA interactions [31],[35]. Arg also seems to have a slightly higher propensity for pyrimidines in the minor groove (4.4 for Cyt and 4.5 for Thy) compared to the major groove (3.4 and 3.3 respectively).  

Lys propensities for both grooves are lower than Arg:

Although Lys has similar electrostatic properties as Arg, we observe relatively smaller propensities for Lys to all bases in the major and minor grooves. This suggests that electrostatic interactions are not the dominant factor in DNA-recognition in the grooves and that Lys probably interacts with the backbone atoms of DNA.

###### Propensity differences between residues in the major groove are much higher than those in the minor groove

The minor groove propensity scores show that except for Arg and to some extent Tyr, most other residues have similar affinity for all mononucleotides (values close to 1). However, in the major groove many residues (for example, Asn preferring Ade with propensity ~5.0, His preferring Gua with propensity ~3.0, both Asp and Glu preferring Cyt with propensity ~2.0) show a greater variation. The lower residue-wise specificity in the minor groove might be an indication of the essential role of structure in protein-DNA recognition in the minor groove. This subsequently implies a greater role of indirect recognition through the DNA-backbone in these interactions.

###### High Arg-Gua propensities in major groove are replaced by Arg-Ade pairs in the minor groove

Whereas Arg-Gua pair does have a very high propensity (>6.0) in the major groove as expected, Arg seems to prefer Ade in the minor groove (propensity>5.0). The propensity of Arg for Ade in the major groove and Gua in the minor groove is lower in comparison (both less than 3.0). This is a significant observation as Arg-Gua has been widely known as a preferred residue-base pair in all protein-DNA interactions [31,35]. Arg also seems to have a slightly higher propensity for pyrimidines in the minor groove (4.4 for Cyt and 4.5 for Thy) compared to the major groove (3.4 and 3.3 respectively).

###### Lys propensities for both grooves are lower than Arg

Although Lys has similar electrostatic properties as Arg, we observe relatively smaller propensities for Lys to all bases in the major and minor grooves. This suggests that electrostatic interactions are not the dominant factor in DNA-recognition in the grooves and that Lys probably interacts with the backbone atoms of DNA.

#### Major and minor groove contact prediction

**Figure 4:**
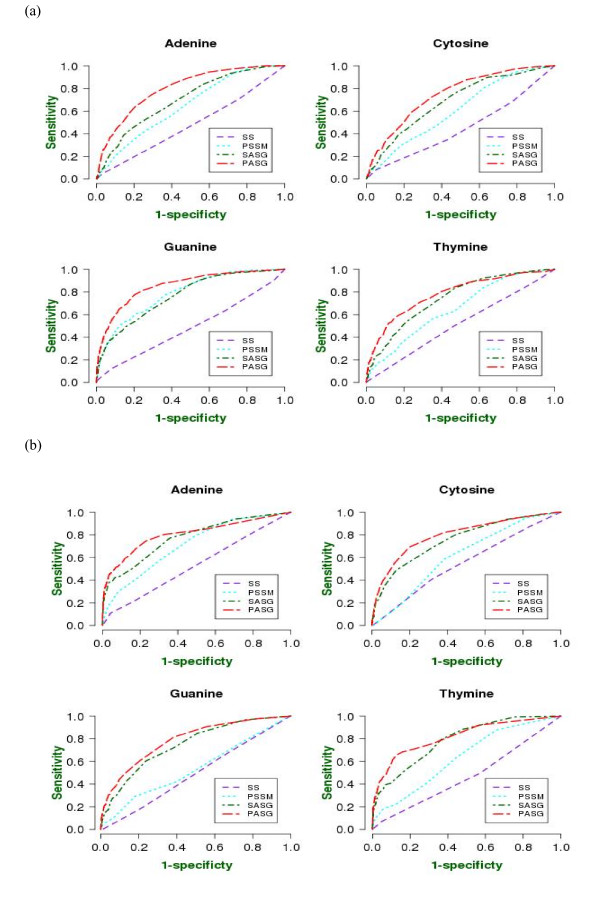
**ROC curves of prediction performance for (a) major and (b) minor groove contacts between amino acid residues and mononucleotides**. Abbreviations as in Figure 2.

Prediction performance of mononucleotide-specific contacts in the major and minor grooves, using single sequences, PSSM and hybrid input features is shown in Figure 4 [for numerical values see Additional file 1: Table S10]. The most striking observation from the prediction results is that the single sequence based predictions are quite poor. For most single sequence predictions, the AUC values are less than 50%, which is expected from a random prediction model. This implies that single sequence information is not sufficient to make significant predictions of the major and minor groove binding, despite the subtle differences in the propensities of individual mononucleotides. The propensity biases shown to exist in the above results are probably off-set by the over-fitting of prediction models during training, thereby leaving the specific binding unpredictable for a strictly cross-validated neural network. The PSSM-based predictions, [see Additional file 1: Table S10], show a significant improvement over the single sequence results. However, the prediction performances are still much poorer than any of the results presented above. As an example, the average AUC for mononucleotide predictions irrespective of the contact type is ~67%, compared to ~47% in the major groove and ~52% in the minor groove predictions. This loss of predictability is significantly compensated by the addition of simple structural information in the form of secondary structure and solvent accessibility. To illustrate, a hybrid predictor using single sequence, solvent accessibility and secondary structure gives an average of ~73% in the major groove and 77% in the minor groove, compared with 75.6% for mononucleotide specific contacts without identifying the contact type. Thus for the major and minor groove contacts, a hybrid feature set using structural information is almost as accurate as the overall mononucleotide contact prediction. This observation proves the importance of structural features for the major and minor groove recognition. Interestingly, a large performance difference is observed even between a hybrid feature set using PSSM and structure and PSSM alone. This implies that the explicit use of structural information is crucial for predictions, instead of the implicit structural information present in the evolutionary profiles.

### Main-chain and side-chain recognition

#### Contact statistics

Contacts are abbreviated as MC (main chain) and SC (side chain) in protein and BB (backbone) and NB (nucleotide base) in DNA and their principal statistics are presented in terms of the propensity scores [see Additional file 1: Table S7(panel(b-e))]. Main observations are discussed below:

A comparison of two key DNA-binding residues viz. Arg and Lys, demonstrates that Arg has the highest propensities for side-chain-nucleotide base (*SC-NB*) contacts, whereas Lys prefers to contact the DNA backbone through its side chain (*SC-BB*) (concluded from the order of propensity scores for the same residue in all four types of contacts). The *SC-NB *contact propensities for Arg are 2–3 times higher than Lys, depicting a clear distinction between the natures of DNA-interactions involving these two residues.

In general, the highest numbers of contacts (for all residues) are observed between the side chain of amino acids and the backbone of nucleotides (*SC-BB*). Although the nucleotides share the same backbone, their propensity scores are different even in this category, apparently due to the sequence-dependent backbone conformation. The overall behavior of residues in this category of contact is similar to the any-atom to any-atom contacts i.e. charged (Lys, Arg and His) and polar (Asn, Gln, Ser, Thr and Tyr) residues preferring to form contacts, whereas most hydrophobic residues having propensities lower than the average. There are fewer contacts between amino acid main chain and DNA-base, presumably due to steric constraints. Quite expectedly Gly has the highest propensity for the *MC-NB *type of contacts and within this category Gly seems to recognize Thy strongly over all the other bases.

#### Prediction performance

**Figure 5:**
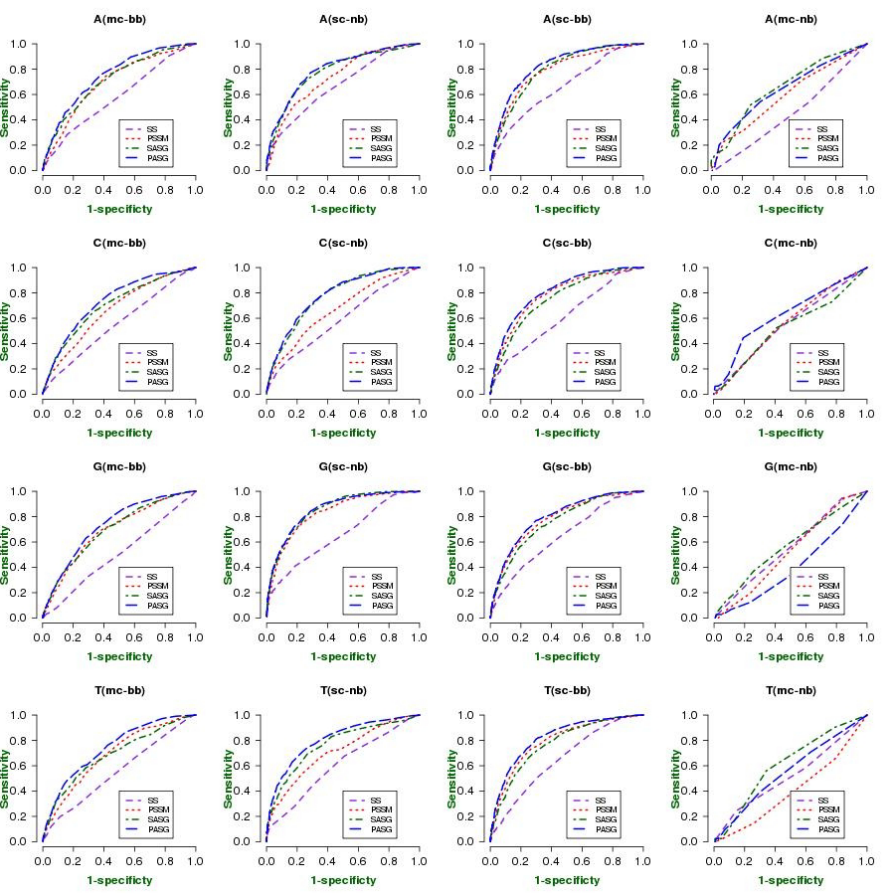
**ROC curves of prediction performance of each contact type between atomic groups viz. amino-acid backbone to nucleotide backbone (MC-BB), amino acid side chain-nucleotide base (SC-NB), amino acid side chain-nucleotide backbone (SC-BB) and amino acid main chain-nucleotide base (MC-NB) of four nucleotides (Ade, Cyt, Gua and Thy) and any amino acid atom to any DNA-atom contact prediction**.

We showed above that the backbone and side chain contacts may not necessarily follow the global patterns of propensity scores. To see which of these contact types are better predicted from sequence and PSSM, a neural network was designed and trained, predicting a 17-bit vector (showing 4 types of contacts for each base discussed above and a single unit showing any to any atom contact between DNA and an amino acid residue). Figure 5 shows ROC graphs of these predictions [For AUC values see Additional file 1: Table S11]. Separate results in each secondary structure are illustrated in Additional file 3: figure S3 and figure S4.

The prediction performance in general is in the following order:

SC-BB > SC-NB > MC-BB > MC-NB

The average PSSM performance for all four base types is 78% for *SC-BB*, 74% for *SC-NB*, 68% for *MC-BB *and just about 52% for *MC-NB *contacts. This result is consistent with the fact that most DNA-protein contacts are formed between the amino acid side chains and the DNA-backbone [39], due to the negatively charged phosphate in DNA and the positively charged side chain of Arg and Lys, which form the most significant contacts with DNA. Hence these contacts are best predicted. The second best category is side chain nucleotide base contacts, which contain the specific information about the identity of the base and the amino acid.

However Gua proves to be an exception to above generalization, with the best prediction performance observed for *SC-NB *contacts. We observed from propensity data that several residues have a different pattern of propensity scores for Gua in their side-chain-nucleotide base contacts. For example, Arg has the highest propensity for Gua in *SC-NB *type of contacts. Even Lys, which typically prefers to interact with Ade, has a higher propensity for Gua in the *SC-NB *contact type. This suggests that Gua recognition is significantly facilitated by *SC-NB *contacts, much in contrast with the other three nucleotides.

PSSM-based predictions are consistently better than single sequences for all contact types and the trend between exposed and buried or in each secondary structure type is more or less the same as those observed in the mononucleotide contacts discussed above.

### Dinucleotide step recognition

#### Contact statistics

It is observed that on an average the number of amino acids forming contacts with a given dinucleotide is about ~1% of all residues [see Additional file 1: Table S5]. Contacts with AG/CT (2.3%), AC/GT (2.2%) and AT/AT (1.7%) are the most abundant and TA/TA (0.3%), GC/GC (0.6%) and GA/TC (0.8%) are scarce. This leaves little data to analyze the individual propensity scores with confidence and hence, we select only the residues most abundant in the binding regions. Table 1 shows the results of such calculations. Key observations from this analysis are discussed in the following.

**Table 1:** Propensity of dinucleotide steps for most significant residues

Step	G	H	K	N	P	Q	R	S	T	W	Y
AA/TT	1.02	1.57	2.37	2.04	0.69	0.91	3.01	1.12	1.26	0.7	1.36
AC/GT	0.94	1.90	2.03	1.84	0.28	1.18	2.80	1.28	1.56	1.58	1.25
AG/CT	1.18	1.60	1.97	1.64	0.40	1.41	2.63	1.42	1.41	1.54	1.55
AT/AT	1.09	1.49	1.92	2.19	0.67	1.17	2.89	1.69	1.3	1.01	1.72
CA/TG	1.00	0.64	1.88	1.26	0.98	0.66	3.64	1.22	1.38	0.81	1.56
CC/GG	1.00	1.91	1.61	1.24	0.40	0.95	2.88	1.73	1.38	1.73	1.27
CG/CG	1.16	1.32	1.84	1.65	0.59	1.45	3.17	1.34	1.20	0.86	1.17
GA/TC	1.69	0.99	1.92	1.74	0.20	0.80	3.79	1.54	1.23	0.64	1.02
GC/GC	0.77	1.63	1.58	1.85	0.69	0.99	3.86	1.56	1.34	1.31	1.59
TA/TA	1.60	2.55	1.94	1.33	1.66	1.55	2.79	0.57	0.54	2.61	1.38

Arg has one of the lowest TA/TA-propensities (among all the dinucleotide steps) whereas Gly and Pro have significantly higher values for the same dinucleotide step. Gly with an absent side chain and Pro with an unusual side chain may interact through their main chain atoms with the minor groove as in the typical TATA-box minor groove binding proteins. This is examined further in the next sections when we look at each groove type propensity.

Although Arg was found to have the highest propensity for Gua, its GG/CC propensity does not follow the same trend. Rather Arg has its highest propensity (3.86) for GC/GC step, which probably allows Arg to bind Gua on both strands of DNA. Such binding would not be possible for the GG/CC dinucleotide step. Similarly, the CG/CG dinucleotide step in Arg may facilitate Gua-contacts on both strands, although its propensity is relatively lower (3.17) than the GC/GC dinucleotide step. It may be added that our non-redundant data contains only one chain of a dimer and all such diagonal contacts-although indirectly observed in propensity scores-are counted only once.

Purine-pyrimidine recognition seems irrelevant as far as Arg is concerned, as one dipurine-dipyrimidine pair (AG/CT) has the lowest Arg propensity (2.63), whereas the other (GA/TC) has among the highest values (3.79). On the other hand CA/TG also has a high propensity for Arg (3.64). All these observations together suggest that the presence of a purine, particularly Gua on both strands in diametric positions facilitate recognition by Arg residues as they can form a geometrically viable dimer-DNA contact.

Three polar residues (Tyr, Ser and Asn) have their highest propensities (1.72, 1.69 and 2.19 respectively) for the same dinucleotide step i.e. AT/AT. In a separate study, we have found that the average solvent accessibility of Thy is much higher than the averages in all other nucleotides [40]. The high propensities of polar residues for AT dinucleotide may be related to the hydrophilic nature of Thymine on both strands. However, the other apparently similar dinucleotide step TA/TA does not show similar behavior, which could be due to conformational requirements of interactions between side chains and dinucleotide steps.

#### Prediction performance

**Figure 6:**
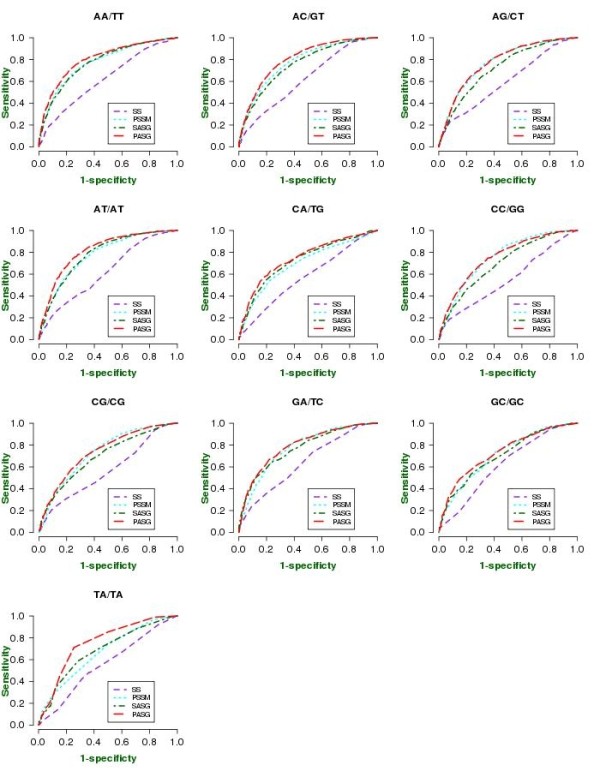
**ROC curves of prediction performance for dinucleotide step contacts of residues**. For all dinucleotide contacts evolutionary profiles show a significant improvement on single sequence-based prediction, which is further improved by adding local structure information i.e. secondary structure (SS) and solvent accessibility (ASA) and global amino-acid composition (GAC).

Figure 6 summarizes the dinucleotide prediction results using single sequences, PSSM and structural information similar to those discussed above [For detailed results see Additional file 1: Table S12]. The dinucleotide step contacts can be predicted with an AUC of nearly 73% using single sequences with structure, and 77% using PSSM with structure. Without using structural information, single sequences show almost no prediction capability (AUC ~60%), whereas PSSM can give ~74% AUC. The prediction performances using structural information, [see Additional file 1: Table S12] for 10 types of dinucleotide steps generally correspond to the amount of available data in each category; AT/AT, AC/GT and AA/TT contacts are best predicted (AUC = 82%, 80% and 80%, respectively) whereas GC/GC and CG/CG contacts are the most difficult to predict (AUC = 73% and 74%, respectively). PSSM based predictions are the lowest for the TA/TA dinucleotide step (AUC = 67% compared with ~77% in most other dinucleotide steps). Apart from few data being available for this dinucleotide, it may also be due to the unusual nature of TA binding to proteins, e.g., in TATA box protein, where, unlike typical protein-DNA interactions, it binds in the minor groove of DNA instead of the major groove [41,42]. This is further supported by the fact that TA-binding residue prediction shows the highest improvement, if local secondary structure and solvent accessibility of the residues are added to the neural network inputs. Moreover, the minor groove contacts of TA/TA are among the best predicted as discussed in the following section.

#### Major and minor groove contact statistics

Dinucleotide step propensities for most significant residue types in the major and minor grooves are listed in Table 2 and comparisons illustrated in Figure 3) Key observations are as follows:

**Table 2:** Propensity of dinucleotide steps in major and minor groove (most significant residues)

Groove	Step	H	K	N	Q	R	Y
Major	AA/TT	1.23	0.64	5.41	3.80	2.21	1.18
	AC/GT	2.71	1.09	2.90	1.21	4.14	1.03
	AG/CT	1.72	1.92	2.54	3.21	2.92	1.48
	AT/AT	2.32	0.65	4.95	1.85	2.55	2.10
	CA/TG	1.35	0.26	2.58	0.56	3.40	1.50
	CC/GG	3.66	1.09	1.49	1.00	4.17	0.77
	CG/CG	1.90	1.78	0.46	2.08	5.36	1.10
	GA/TC	2.58	2.83	1.03	0.80	5.14	0.38
	GC/GC	2.34	3.03	1.32	1.08	5.03	0.70
	TA/TA	2.26	0.00	6.48	4.72	0.72	3.19
Minor	AA/TT	1.28	1.32	0.45	0.42	8.15	0.00
	AC/GT	1.92	1.97	0.00	0.94	3.53	3.17
	AG/CT	0.00	0.63	2.64	1.86	3.00	1.79
	AT/AT	0.52	1.51	2.66	0.30	3.75	2.38
	CA/TG	0.00	1.13	1.38	1.33	5.79	1.80
	CC/GG	1.91	1.80	2.35	1.49	3.39	2.27
	CG/CG	0.00	2.19	2.41	2.73	2.25	2.38
	GA/TC	0.00	1.26	0.75	2.38	3.62	0.00
	GC/GC	0.00	2.56	1.35	1.92	2.67	1.23
	TA/TA	0.00	3.88	0.00	0.00	4.94	3.65

##### Diversity of propensities in the minor groove is higher than mononucleotides

There is a greater variation in propensity values for residue-dinucleotide steps, in comparison to the mononucleotide preferences shown above. For example, Arg has a clear preference for AA/TT step in the minor groove (propensity = 8.15, similar to but much higher than Arg-Ade preference in mononucleotides, propensity = 5.32). Similarly, Arg is found to have a preference for the CA/TG step (propensity = 5.79, higher than any mononucleotide propensity for Arg in the minor groove). Thus, the dinucleotide step recognition seems to be much stronger than mononucleotide recognition in the minor groove.

##### Asn has a high propensity for TA/TA in the major but not the minor groove

TA/TA, AA/TT and AT/AT dinucleotide steps show high propensities for Asn in the major groove. No such preference is observed in the minor groove.

In addition, His shows a strong preference for CC/GG dinucleotide step in the major groove and a subtle preference in the minor groove. In general, we can conclude that there are some clear and some subtle preferences of single residues to form contacts with either or both the major and minor grooves of dinucleotide steps and these preferences could be exploited for the prediction of specific contacts. Results of such efforts are presented in the following section.

#### Major and minor groove contact predictions

**Figure 7:**
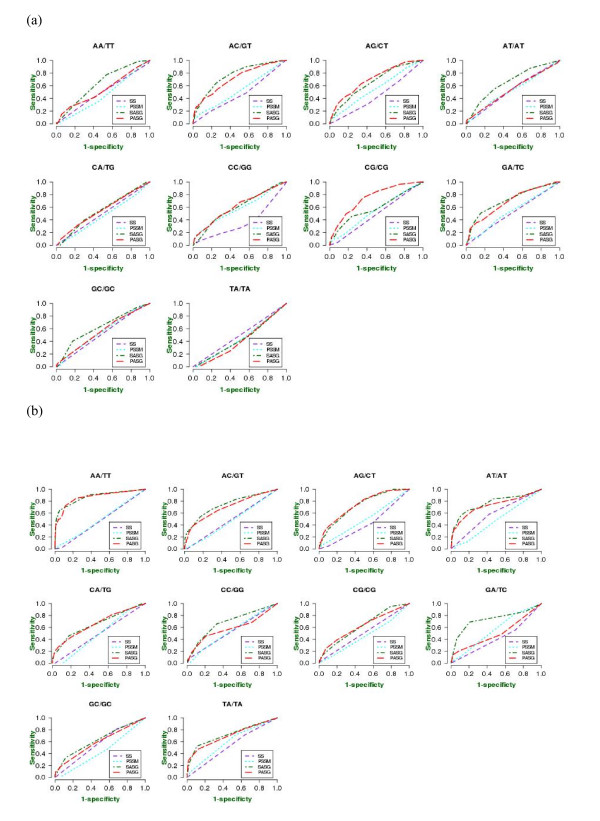
**ROC curves of prediction performance for (a) major and (b) minor groove contacts between amino acid residues and dinucleotide steps**.

Since the dinucleotide step contacts in each category are fewer than any of the contact types discussed so far, the prediction performance was somewhat erratic, as neural networks stopped either too early in the cross-validation scheme or over-fitted the training model. Some observations are nonetheless made, which can only be confirmed when a sufficiently large number of complexes is available. The results for the major and minor groove contact prediction are shown in Figures 7a and 7b [detailed values in Additional file 1: Table S13]. Similar to mononucleotide contacts, the dinucleotide step contact prediction is very poor without structural information, the use of which significantly improves the situation. The best predictions are obtained for AA/TT step contacts in the minor groove (~88% AUC). The TA/TA contact prediction performs much better in the minor groove than in the major groove, consistent with the known behavior of this pattern of binding in the minor groove.

### Online prediction web server

It is clear from the above results that mononucleotide and dinucleotide step contact predictions can be performed effectively using single sequences and PSSM. The major and minor groove contacts strongly depend on the structure and therefore, sequence-based predictions cannot be performed. We developed a web server to predict mononucleotide and dinucleotide step specific contacts using PSSM inputs. The web server is based on the parameters trained on all 159 proteins using a five-residue window to avoid over-fitting and also to take advantage of all available proteins. Apart from contact prediction scores for all four mononucleotides and 10 dinucleotide steps, the web server also returns multiple alignments used for the prediction. This web server is freely available at  or .

## Conclusion

We have shown that mononucleotide- and dinucleotide-specific contacts can be predicted from single amino acid sequences, evolutionary profiles and basic structural information with almost the same accuracy as general DNA-binding sites. However, the major and minor groove contacts strongly depend on the structure and cannot be predicted from sequence alone. The best prediction performance of sequence-based prediction is ~80%, whereas the best major/minor groove dinucleotide step prediction could reach as high as 87%, measured by the area under the ROC graph. This study will help us better understand and predict the specific base-amino acid interactions in protein-DNA complexes.

## Authors' contributions

All calculations were performed by MA, under SA's supervision. SA conceived of, designed and coordinated the study. AS and KM contributed in finalizing the scope and design. SA, KM and MA prepared the manuscript. All authors read and approved the manuscript.

## Supplementary Material

Additional file 1 **Detailed results of data statistics, propensity and predictions in tabular format.** Click here for file

Additional file 2 **Results of t-test and chi-squared tests on the contact data and prediction scores.** Click here for file

Additional file 3 **Additional results of propensity and predictions in graphical format.** Click here for file
