# Supplementary material for: Prediction of mono- and di-nucleotide-specific DNA-binding sites in proteins using neural networks
Source: BMC Struct Biol. 2009 May 13;9:30. doi: 10.1186/1472-6807-9-30 (PMC2693520; doi:10.1186/1472-6807-9-30)
Supplement: Additional file 1 — Detailed results of data statistics, propensity and predictions in tabular format. [file 1472-6807-9-30-S1.pdf]

Table of Contents

Data set and contacting residue counts: 2
TableS1: PDB codes of PDNA-159, a non-redundant data set of DNA-binding proteins. 2
TableS2: Frequency distribution of mononucleotide specific contacts of residues in different secondary structures and states of solvent accessibility. 3
TableS3: Frequency distribution of major and minor groove contacts of mononucleotides. 3
TableS4: Frequency distribution of different contact types of residues in different secondary structures and states of solvent accessibility. 4
TableS5: Frequency distribution of dinucleotide step-specific contacts of single residues for different secondary structures and states of solvent accessibility. 5
TableS6: Frequency distributions of major and minor groove contacts of dinucleotide steps. 6
Propensity scores: 7
TableS7: Propensity scores for different contact types of mononucleotides. 7
TableS8: Propensity of amino acid residue for major and minor groove contacts with mononucleotides. 8
Prediction performance: 9
TableS9: Prediction performance of mononucleotide contacts. 9
TableS10: Prediction performance for major and minor groove contact of mononucleotides 10
TableS11: Prediction performance for different contact types of mononucleotides. 11
TableS12: Prediction performance for dinucleotide step contacts. 12
TableS13: Prediction performance for major and minor groove contacts of dinucleotide steps.... 13

Data set and contacting residue counts:

TableS1: PDB codes of PDNA-159, a non-redundant data set of DNA-binding proteins. (Fifth letter is chain identifier).

**Helix Turn Helix (Group I)**										
1k78I	1lliB	1ignA	2heoD	1f4kB	1tc3C	1d3uB	1l3lB	1j59A	1hcrA	1k79D
1mnmC	1b8iB	1j1vA	2irfJ	1ig7A	1pufB	1bl0A	1yrnA	1au7B	6paxA	1akhB
1k61B	1hlvA	1pufA	2o4aA	1gxpB	1trrG	1sfuB	1pueF	1w0uA	1qbjB	
**Zinc Coordinating (Group II)**										
1f2iG	2nllB	1ubdC	2a66A	2i13B	1hwtH	1llmD	1zmeD	2ahiA	1p47A	1v15D
**Zipper type (Group III)**										
1am9B	1h89C	1h8aA	1nlwD	2h7hB	1gd2G	1nlwB				
**Other alpha helical (Group IV)**										
1crxB	1n6jB	1zs4C	1cktA	1b3tA	1mnmB	1qrvA				
**Beta-Sheet (Group V)**										
1qn7B	1rm1C									
**Beta-hairpin/ Ribbon (Group VI)**										
1bdtC	1xbrB	1mjqG	2iieA	1ihfA	2i06A	2bnwA	2ntcB			
**Enzymes (Group VII)**										
1a35A	1m5rB	1eyuB	1ku7A	1tezB	2dtuC	1sa3B	1r7mB	1iawB	1qssA	2aq4A
1ippA	1k82D	1dizA	4bdpA	1skrA	2ex5A	1zetA	1rh6B	3bamA	2p0jB	2oaaA
1stxA	1qumA	1d02B	1mowD	1rrqA	2ezvA	2i3pB	1rxwA	1qaiB	2aoqA	1xhvB
2pi4A	1rrjA	1dfmB	1murA	1wbdA	2fccB	2ofiA	2owoA	1emjA	2bcqA	1t2tA
1cw0A	1ewqA	1f6oA	1oe5A	1wteA	2fkcB	2b9sB	2h27D	2ge5A	1dnkA	1n56B
1dc1A	1fjxA	1fiuD	1orpA	1ya6A	1m5xB	2dp6A	2is6B	2jg3D	1oupA	2g1pA
1yqmA	2q10A									
**Histones/ Histone-like (Group VIII)**										
1wd1A	1kx5E	1eqzG	1kx5C	2cv5A	1kx5F	1kx5D	2cv5C	2cv5D		
**Others/ Unknown (Group IX)**										
1j3eA	1je8B	1jeyA	1jeyB	1jj4A	1lq1D	1nfkB	1ozjA	2d5vB	2eviA	2iszB
1u8bA	1uutB	2a07J	2c6yA							

TableS2: Frequency distribution of mononucleotide specific contacts of residues in different secondary structures and states of solvent accessibility. All percentages are calculated relative to the total number of residues in corresponding categories (column labels). For example, there are 3.15% residues in contact with Ade, whereas within helical residues this number is 2.27%.

	Percent contacts (absolute number)	Helix	Strand	Others	Exposed	Buried
Ade	3.15 (1018)	2.27 (303)	3.11 (156)	4.00 (559)	4.85 (785)	1.44 (233)
Cyt	3.06 (991)	2.45 (327)	2.47 (124)	3.86 (540)	4.61 (745)	1.52 (246)
Gua	3.66 (1185)	2.92 (390)	3.93 (197)	4.28 (598)	5.47 (885)	1.85 (300)
Thy	3.35 (1084)	2.90 (388)	2.87 (144)	3.95 (552)	5.24 (847)	1.46 (237)

TableS3: Frequency distribution of major and minor groove contacts of mononucleotides. All values are in % units and absolute numbers are in parenthesis.

Groove and nucleotide		Overall	Helix	Strand	Others
Major	Ade	0.52 (167)	0.05 (70)	0.54 (27)	0.50 (70)
	Cyt	0.62 (202)	0.58 (78)	0.68 (34)	0.64 (90)
	Gua	0.92 (297)	0.88 (117)	1.14 (57)	0.88 (123)
	Thy	0.64 (208)	0.82 (110)	0.56 (28)	0.50 (70)
Minor	Ade	0.36 (118)	0.12 (16)	0.28 (14)	0.63 (88)
	Cyt	0.24 (78)	0.15 (20)	0.12 (6)	0.37 (52)
	Gua	0.37 (119)	0.21 (28)	0.26 (13)	0.56 (78)
	Thy	0.43 (140)	0.20 (27)	0.44 (22)	0.65 (91)

TableS4: Frequency distribution of different contact types of residues in different secondary structures and states of solvent accessibility. All values are in % units and absolute numbers are in parenthesis. Abbreviations: MC: Protein main chain, SC: Protein side chain, BB: DNA backbone, NB: Nucleotide base

Contact type and nucleotide		Overall	Helix	Strand	Others	Exposed	Buried
MC-BB	Ade	0.92 (297)	0.35 (47)	0.88 (44)	1.47 (206)	1.40 (226)	0.44 (71)
	Cyt	0.98 (318)	0.43 (58)	0.82 (41)	1.57 (219)	1.42 (229)	0.55 (89)
	Gua	0.99 (320)	0.47 (63)	0.80 (40)	1.55 (217)	1.39 (225)	0.59 (95)
	Thy	0.99 (320)	0.55 (73)	0.58 (29)	1.56 (218)	1.49 (241)	0.49 (79)
SC-NB	Ade	0.74 (241)	0.58 (78)	0.72 (36)	0.91 (127)	1.35 (218)	0.14 (23)
	Cyt	0.70 (227)	0.67 (90)	0.68 (34)	0.74 (103)	1.21 (195)	0.20 (32)
	Gua	1.07 (346)	0.98 (131)	1.30 (65)	1.07 (150)	1.89 (305)	0.25 (41)
	Thy	0.73 (235)	0.59 (79)	0.72 (36)	0.86 (120)	1.30 (211)	0.15 (24)
SC-BB	Ade	1.96 (634)	1.50 (201)	2.05 (103)	2.36 (330)	2.99 (483)	0.93 (151)
	Cyt	1.79 (580)	1.64 (219)	1.36 (68)	2.10 (293)	2.69 (435)	0.90 (145)
	Gua	2.06 (667)	1.84 (246)	2.13 (107)	2.25 (314)	2.95 (477)	1.17 (190)
	Thy	2.01 (650)	2.01 (268)	1.79 (90)	2.09 (292)	2.98 (482)	1.04 (168)
MC-NB	Ade	0.11 (35)	0.04 (5)	0.10 (5)	0.18 (25)	0.19 (31)	0.02 (4)
	Cyt	0.19 (63)	0.04 (5)	0.16 (8)	0.36 (50)	0.33 (54)	0.06 (9)
	Gua	0.23 (73)	0.09 (12)	0.16 (8)	0.38 (53)	0.37 (60)	0.08 (13)
	Thy	0.13 (41)	0.06 (8)	0.10 (5)	0.20 (28)	0.24 (39)	0.01 (2)

TableS5: Frequency distribution of dinucleotide step-specific contacts of single residues for different secondary structures and states of solvent accessibility. All values are in % units and absolute numbers are in parenthesis.

Step	Overall	Helix	Strand	Others	Exposed	Buried
AA/TT	1.50 (485)	1.14 (152)	1.24 (62)	1.94 (271)	2.36 (382)	0.64 (103)
AC/GT	2.22 (719)	1.98 (264)	1.93 (97)	2.56 (358)	3.38 (546)	1.07 (173)
AG/CT	2.34 (758)	1.60 (214)	2.61 (131)	2.96 (413)	3.45 (558)	1.24 (200)
AT/AT	1.66 (538)	1.29 (173)	1.59 (80)	2.04 (285)	2.60 (421)	0.72 (117)
CA/TG	0.78 (253)	0.58 (78)	0.76 (38)	0.98 (137)	1.28 (207)	0.28 (46)
CC/GG	1.60 (517)	1.44 (193)	1.32 (66)	1.85 (258)	2.45 (397)	0.74 (120)
CG/CG	1.22 (394)	1.03 (138)	1.24 (62)	1.39 (194)	1.75 (283)	0.69 (111)
GA/TC	0.75 (244)	0.58 (77)	0.68 (34)	0.95 (133)	1.18 (191)	0.33 (53)
GC/GC	0.56 (180)	0.53 (71)	0.42 (21)	0.63 (88)	0.83 (134)	0.28 (46)
TA/TA	0.30 (97)	0.22 (30)	0.12 (6)	0.44 (61)	0.51 (83)	0.09 (14)

TableS6: Frequency distributions of major and minor groove contacts of dinucleotide steps. All values are in % units and absolute numbers are in parenthesis.

Groove	Step	Overall	Helix	Strand	Others
Major	AA/TT	0.20 (65)	0.26 (35)	0.14 (7)	0.16 (23)
	AC/GT	0.46 (148)	0.58 (77)	0.60 (30)	0.29 (41)
	AG/CT	0.42 (136)	0.32 (43)	0.58 (29)	0.46 (64)
	AT/AT	0.25 (82)	0.30 (40)	0.20 (10)	0.23 (32)
	CA/TG	0.12 (38)	0.14 (19)	0.04 (2)	0.12 (17)
	CC/GG	0.38 (123)	0.39 (52)	0.52 (26)	0.32 (45)
	CG/CG	0.32 (105)	0.23 (31)	0.46 (23)	0.36 (51)
	GA/TC	0.20 (65)	0.26 (35)	0.22 (11)	0.14 (19)
	GC/GC	0.11 (37)	0.13 (17)	0.04 (2)	0.13 (18)
	TA/TA	0.05 (16)	0.09 (12)	0.00 (0)	0.03 (4)
Minor	AA/TT	0.15 (50)	0.04 (6)	0.04 (2)	0.30 (42)
	AC/GT	0.14 (45)	0.05 (7)	0.14 (7)	0.22 (31)
	AG/CT	0.21 (69)	0.11 (15)	0.22 (11)	0.31 (43)
	AT/AT	0.23 (74)	0.09 (12)	0.24 (12)	0.36 (50)
	CA/TG	0.11 (37)	0.05 (7)	0.14 (7)	0.16 (23)
	CC/GG	0.14 (44)	0.12 (16)	0.06 (3)	0.18 (25)
	CG/CG	0.13 (41)	0.10 (13)	0.02 (1)	0.16 (23)
	GA/TC	0.09 (29)	0.04 (5)	0.02 (1)	0.16 (23)
	GC/GC	0.06 (21)	0.05 (7)	0.06 (3)	0.08 (11)
	TA/TA	0.06 (21)	0.02 (3)	0.02 (1)	0.12 (17)

Propensity scores:

TableS7: Propensity scores for different contact types of mononucleotides.

**(a) Propensity for any mononucleotide-atom to any residue-atom contact**																				
	**A**	**C**	**D**	**E**	**F**	**G**	**H**	**I**	**K**	**L**	**M**	**N**	**P**	**Q**	**R**	**S**	**T**	**V**	**W**	**Y**
Ade	0.4	0.57	0.42	0.21	0.58	0.92	1.51	0.4	2.22	0.17	0.48	2.34	0.53	1.49	2.85	1.28	1.16	0.25	0.95	1.68
Cyt	0.36	0.35	0.96	0.53	0.45	1.09	1.23	0.45	1.96	0.19	0.92	1.69	0.4	1.06	2.87	1.31	1.31	0.33	1.38	1.41
Gua	0.27	0.38	0.66	0.32	0.53	1.08	1.9	0.34	1.7	0.24	0.62	1.47	0.45	1.13	3.64	1.45	1.35	0.29	0.94	1.28
Thy	0.54	0.27	0.21	0.29	0.66	1.12	1.46	0.3	1.98	0.27	0.51	1.68	0.67	1.06	2.81	1.53	1.4	0.41	1.71	1.5
**(b) Propensity for amino-acid backbone to nucleotide backbone contact (MC-BB)**																				
Ade	1.15	0.7	0.39	0.23	0.33	2.69	0.55	0.56	1.63	0.21	0.79	1.3	0.73	0.56	1.8	1.68	1.88	0.54	0.2	1.15
Cyt	0.8	0.24	0.85	0.23	0.47	2.74	0.75	0.79	1.43	0.23	0.87	1.39	0.52	0.96	1.44	2.04	1.48	0.55	1.27	0.89
Gua	0.65	0.49	1.13	0.34	0.75	2.93	1.14	0.57	1.00	0.66	0.17	1.17	0.35	0.99	1.24	2.00	1.53	0.76	0.99	0.55
Thy	1.45	0.22	0.23	0.28	0.48	3.16	1.15	0.47	1.29	0.49	0.57	0.84	0.92	0.92	1.19	2.1	1.39	0.53	0.4	0.6
**(c)Propensity for amino-acid side chain to nucleotide backbone contact (SC-BB)**																				
Ade	0.24	0.37	0.38	0.15	0.69	0.00	1.84	0.46	2.75	0.11	0.41	2.09	0.44	1.19	3.27	1.49	1.21	0.18	1.33	1.89
Cyt	0.22	0.15	0.7	0.2	0.28	0.00	1.22	0.42	2.67	0.17	1.3	1.69	0.44	1.28	3.34	1.38	1.64	0.24	1.17	1.75
Gua	0.23	0.35	0.64	0.38	0.34	0.00	1.86	0.37	1.69	0.26	0.65	1.66	0.65	1.15	3.64	1.59	1.93	0.21	1.24	1.73
Thy	0.33	0.21	0.16	0.17	0.83	0.00	1.57	0.19	2.34	0.23	0.41	1.77	0.65	1.05	3.14	1.52	1.85	0.39	2.21	1.91
**(d) Propensity for amino-acid backbone to nucleotide side chain contact (MC-NB)**																				
Ade	0.34	0.00	0.00	0.00	0.00	4.41	0.97	0.61	0.00	0.82	0.00	1.56	1.39	1.03	3.43	1.07	1.24	0.44	0.00	0.74
Cyt	0.63	0.00	1.11	0.2	1.42	4.5	1.91	0.00	1.09	0.19	0.00	2.4	0.41	0.67	1.14	1.45	0.71	0.24	1.04	1.28
Gua	0.16	0.00	0.83	0.17	1.36	5.57	0.00	0.43	0.93	0.26	1.97	1.58	0.32	0.9	0.81	1.75	0.64	0.5	1.33	1.4
Thy	0.63	0.00	0.8	0.00	0.00	6.85	0.00	0.39	1.66	0.24	2.24	1.09	0.63	0.00	1.1	0.43	0.53	0.99	1.17	0.75
**(e)Propensity for amino-acid side chain to nucleotide side chain contact (SC-NB)**																				
Ade	0.06	0.56	0.42	0.33	0.43	0.00	1.17	0.13	1.42	0.31	0.42	4.15	0.38	2.91	4.13	1.03	0.39	0.19	0.00	1.68
Cyt	0.06	0.81	1.68	1.59	0.41	0.00	1.9	0.33	0.83	0.00	0.67	2.32	0.21	1.21	4.06	0.82	0.8	0.29	1.81	1.58
Gua	0.03	0.35	0.41	0.17	0.42	0.00	2.62	0.19	2.16	0.05	0.58	1.82	0.16	1.46	5.77	1.22	0.61	0.11	0.41	0.94
Thy	0.17	0.33	0.00	0.42	0.97	0.00	1.03	0.23	1.66	0.12	0.94	2.38	0.84	1.14	4.81	0.91	0.73	0.28	1.63	2.17

TableS8: Propensity of amino acid residue for major and minor groove contacts with mononucleotides.

Groove and nucleotide		Amino acid residue														
		D	E	F	G	H	K	M	N	P	Q	R	S	T	W	Y
Major	Ade	0.50	0.47	0.48	0.21	1.19	0.88	0.28	4.98	0.52	3.81	2.77	0.98	0.84	0.00	2.29
	Cyt	2.06	1.86	0.54	0.72	2.06	0.48	0.76	2.24	0.13	0.82	3.36	1.10	0.92	1.63	1.30
	Gua	0.14	0.19	0.33	0.68	2.95	2.23	0.68	1.23	0.00	1.43	6.09	1.26	0.50	0.44	0.56
	Thy	0.25	0.63	0.77	0.52	1.67	1.18	0.30	2.83	0.32	1.77	3.30	1.70	1.38	1.46	1.52
Minor	Ade	0.35	0.14	0.40	1.13	1.26	1.65	0.48	1.90	0.86	0.90	5.32	1.07	0.33	0.00	0.68
	Cyt	0.23	0.00	0.83	1.69	1.23	1.70	0.00	1.90	0.60	1.69	4.41	0.00	0.52	1.11	3.05
	Gua	1.35	0.15	1.38	1.63	0.30	1.20	1.27	2.35	0.83	1.84	2.41	0.88	0.91	1.92	2.18
	Thy	0.14	0.22	1.23	1.48	0.00	1.92	1.80	1.50	1.06	0.49	4.55	0.40	0.27	1.51	2.55

Prediction performance:

TableS9: Prediction performance of mononucleotide contacts. All values are percentage area under the curve (AUC) of ROC curves.

Abbreviations: ASA: Accessible surface area, SS: Secondary structure, GAC: Global amino acid composition

Nucleotide	Sequence	PSSM	Sequence +ASA+ SS+GAC	PSSM+ASA+ SS+GAC
Ade	68.4	76.3	76.8	81.0
Cyt	68.3	73.1	73.4	77.7
Gua	68.8	75.8	76.5	80.2
Thy	66.2	75.9	75.9	81.4

TableS10: Prediction performance for major and minor groove contact of mononucleotides. All values are percentage area under the curve (AUC) of ROC curves.

Abbreviations: ASA: Accessible surface area, SS: Secondary structure

Groove	Nucleotide	Sequence	PSSM	Sequence+ASA+SS	PSSM+ASA+SS
Major	Ade	47.8	64.7	70.0	80.0
	Cyt	45.0	61.1	68.9	74.9
	Gua	48.6	78.6	76.7	85.5
	Thy	51.5	65.3	74.5	78.2
Minor	Ade	53.3	71.0	77.4	80.6
	Cyt	55.1	61.5	75.8	80.0
	Gua	49.4	53.8	74.7	78.7
	Thy	47.1	63.6	78.9	82.0

TableS11: Prediction performance for different contact types of mononucleotides. All values are percentage area under the curve (AUC) of ROC curves. Abbreviations: ASA: Accessible surface area, SS: Secondary structure, GAC: Global amino acid composition

Contact types and nucleotide name		Sequence	PSSM	Sequence+ASA +SS+GAC	PSSM+ASA+ SS+GAC
MC-BB	Ade	58.1	69.7	71.5	74.9
	Cyt	55.4	66.3	70.1	73.3
	Gua	50.4	69.0	69.5	73.1
	Thy	55.5	69.0	68.7	74.2
SC-NB	Ade	67.1	73.6	78.1	79.6
	Cyt	58.9	66.9	77.4	78.1
	Gua	65.6	83.0	85.3	85.5
	Thy	60.6	70.9	76.8	80.9
SC-BB	Ade	66.0	78.7	79.5	82.7
	Cyt	61.3	78.3	75.6	80.9
	Gua	64.5	78.0	76.1	79.8
	Thy	65.1	79.3	77.3	82.2
MC-NB	Ade	44.8	59.4	66.1	64.3
	Cyt	54.9	56.1	52.8	62.9
	Gua	55.7	52.3	56.8	40.0
	Thy	52.3	40.3	61.3	55.5
	any2any	68.2	78.3	77.0	82.4

TableS12: Prediction performance for dinucleotide step contacts. All values are percentage area under the curve (AUC) of ROC curves. Abbreviations: ASA: Accessible surface area, SS: Secondary structure, GAC: Global amino acid composition

Step	Sequence	PSSM	Seq+ASA+SS+ +GAC	PSSM+ASA+SS +GAC
AA/TT	62.3	77.4	77.5	80.4
AC/GT	62.1	77.6	75.6	80.4
AG/CT	60.0	77.5	72.5	77.6
AT/AT	61.9	77.3	78.1	81.6
CA/TG	57.3	69.5	72.6	74.6
CC/GG	55.1	75.4	70.0	75.5
CG/CG	56.6	73.2	69.5	73.7
GA/TC	63.9	77.4	76.8	79.1
GC/GC	62.0	71.4	70.6	73.4
TA/TA	55.9	67.0	68.5	75.6

TableS13: Prediction performance for major and minor groove contacts of dinucleotide steps. All values are percentage area under the curve (AUC) of ROC curves.

Abbreviations: ASA: Accessible surface area, SS: Secondary structure

Groove	Step	Sequence	PSSM	Seq+ASA+SS	PSSM+ASA+SS
Major	AA/TT	50.1	45.6	63.6	54.0
	AC/GT	47.1	53.0	76.3	71.6
	AG/CT	44.3	55.4	65.9	70.6
	AT/AT	53.2	53.1	66.3	53.9
	CA/TG	50.1	46.2	55.1	55.3
	CC/GG	36.2	58.1	60.1	61.4
	CG/CG	48.9	54.4	59.1	74.7
	GA/TC	51.4	53.7	71.4	68.9
	GC/GC	52.4	55.0	62.7	56.0
	TA/TA	49.7	44.6	43.8	41.8
Minor	AA/TT	47.1	48.9	87.4	86.7
	AC/GT	48.6	47.0	74.1	69.9
	AG/CT	41.3	49.3	72.6	74.1
	AT/AT	58.0	47.7	76.9	72.9
	CA/TG	48.9	48.5	65.8	66.0
	CC/GG	53.7	54.0	68.3	58.2
	CG/CG	50.8	45.4	62.8	62.3
	GA/TC	42.1	55.8	73.7	50.3
	GC/GC	56.4	43.6	63.0	59.2
	TA/TA	53.7	60.0	72.4	70.5
