# Supplementary material for: Prediction of mono- and di-nucleotide-specific DNA-binding sites in proteins using neural networks
Source: BMC Struct Biol. 2009 May 13;9:30. doi: 10.1186/1472-6807-9-30 (PMC2693520; doi:10.1186/1472-6807-9-30)

Table of Contents

FigureS1: Precision-recall curves for mononucleotide contact prediction using various inputs.. 2

FigureS2: Precision-recall curve for the prediction of side-chain/main-chain classified contacts in mono-nucleotides. 3

FigureS3: Secondary structure specific prediction performance of different residue-mononucleotide contact types 4

FigureS4: Prediction performance for different contact types in exposed and buried regions. .. 5

FigureS5: Precision-recall curves for the prediction of dinucleotide step contact. 6

FigureS6: Dinucleotide step contact prediction in various secondary structures. 7

Figure S7: Dinucleotide step contact prediction in exposed and buried residues........ 8

FigureS1: Precision-recall curves for mononucleotide contact prediction using various inputs.

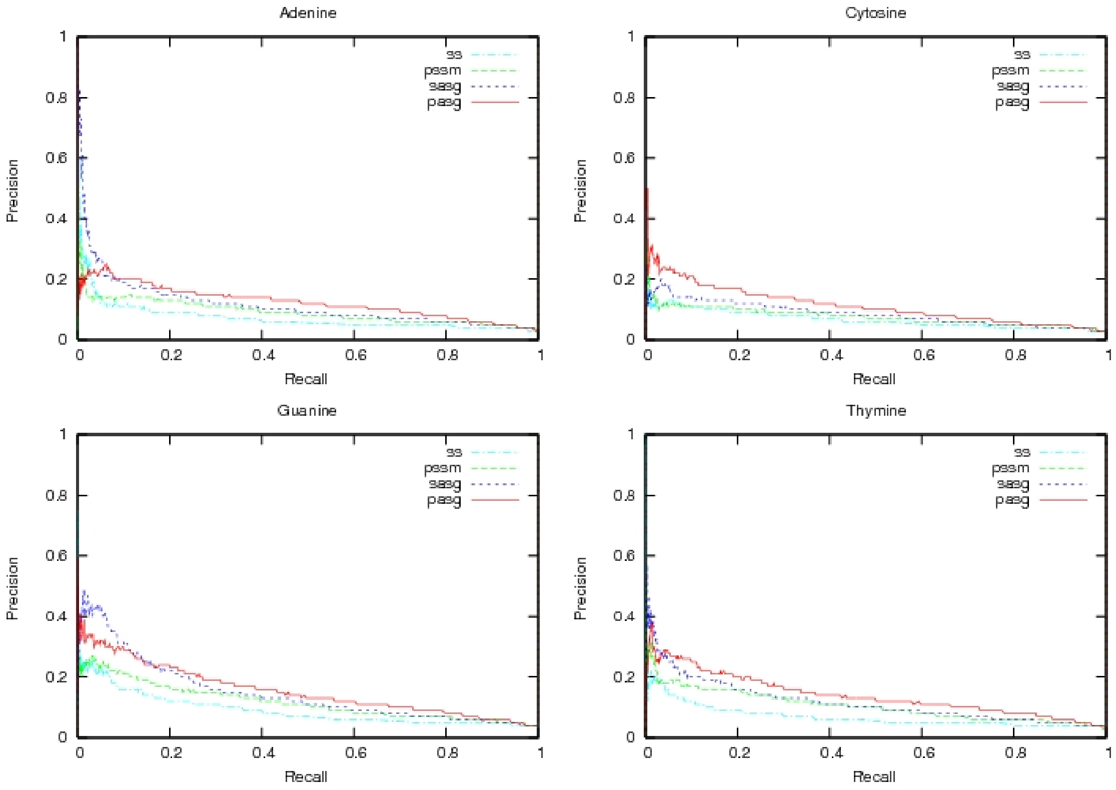

FigureS2: Precision-recall curve for the prediction of side-chain/main-chain classified contacts in mono-nucleotides.

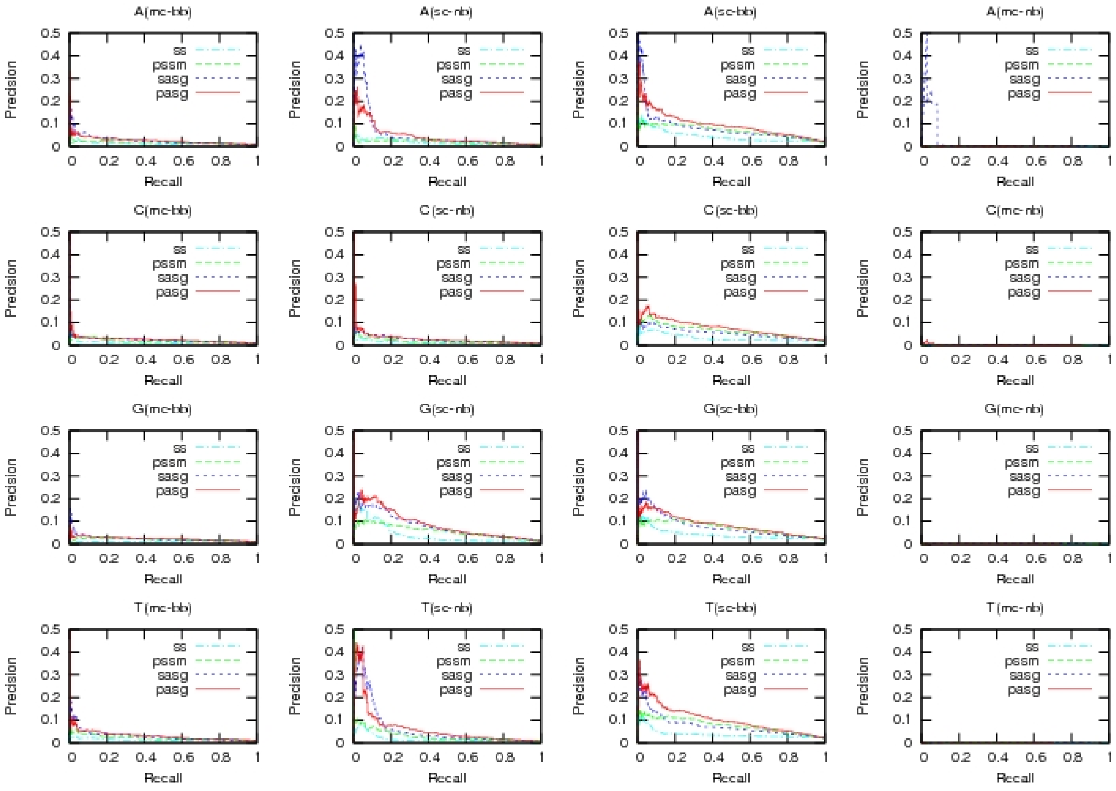

FigureS3: Secondary structure specific prediction performance of different residue-mononucleotide contact types

(a)

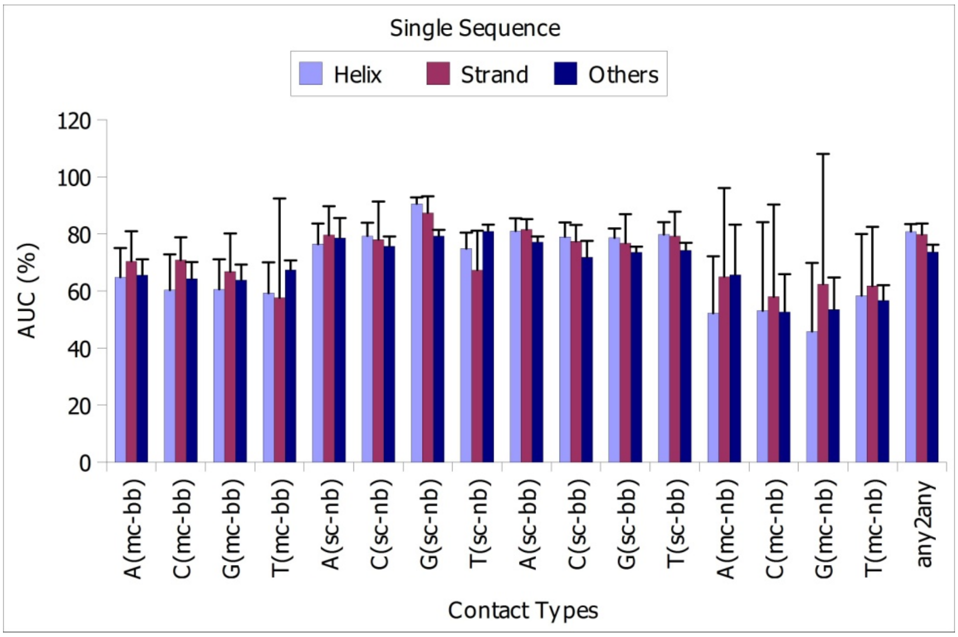

(b)

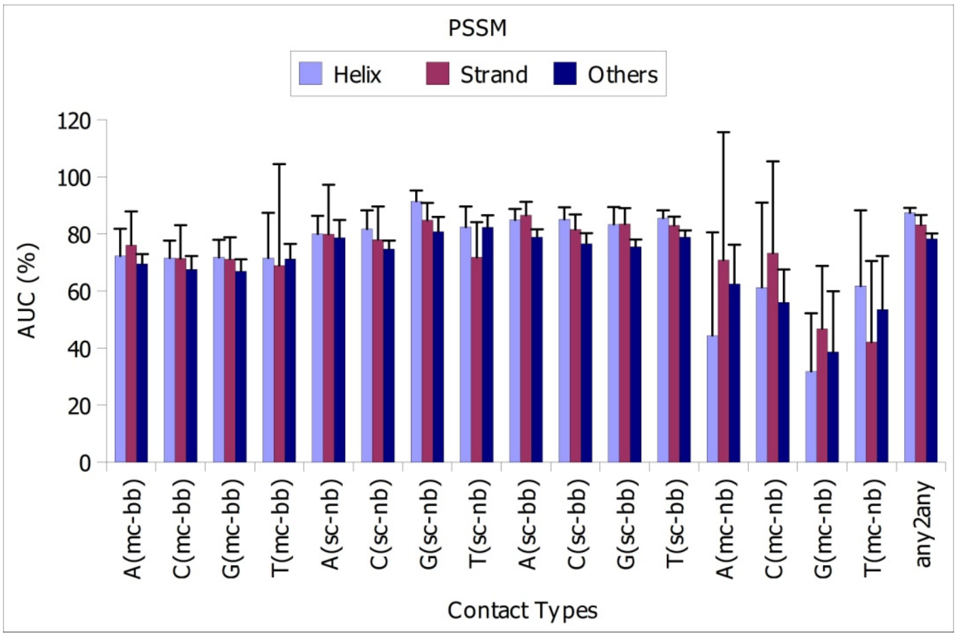

FigureS4: Prediction performance for different contact types in exposed and buried regions.

(a)

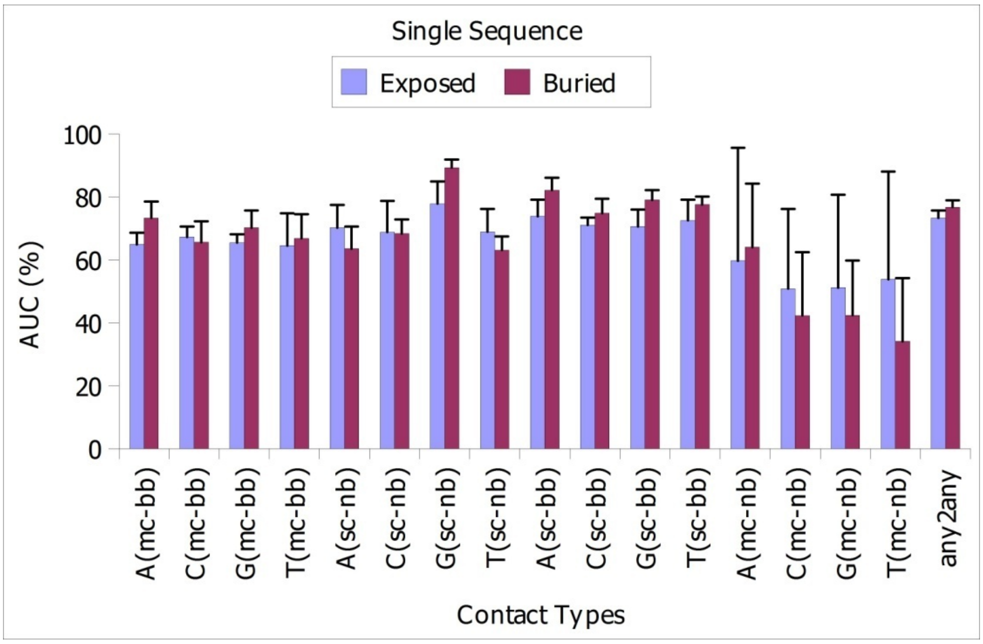

(b)

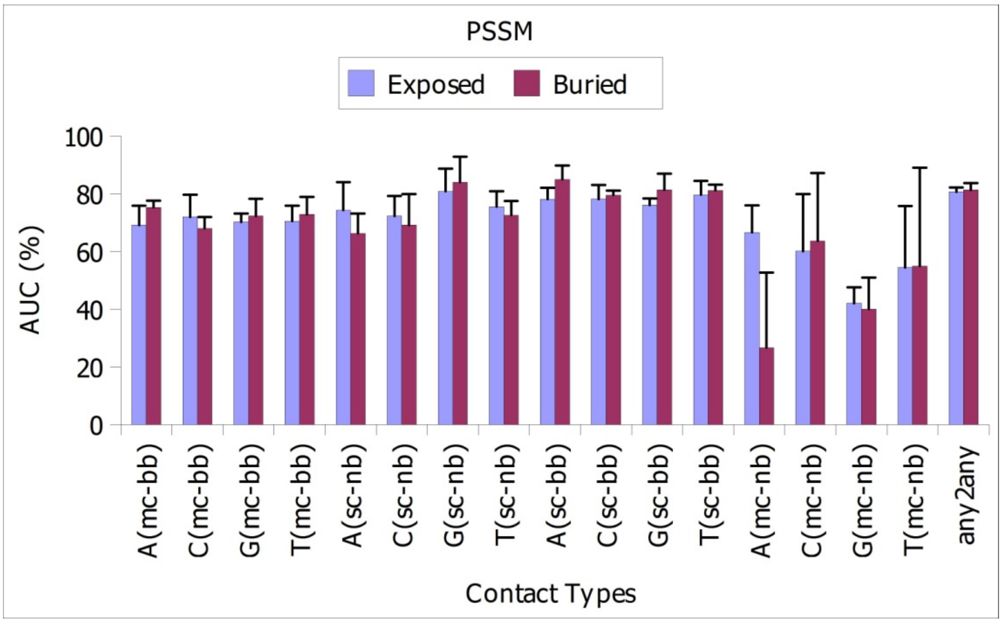

FigureS5: Precision-recall curves for the prediction of dinucleotide step contact.

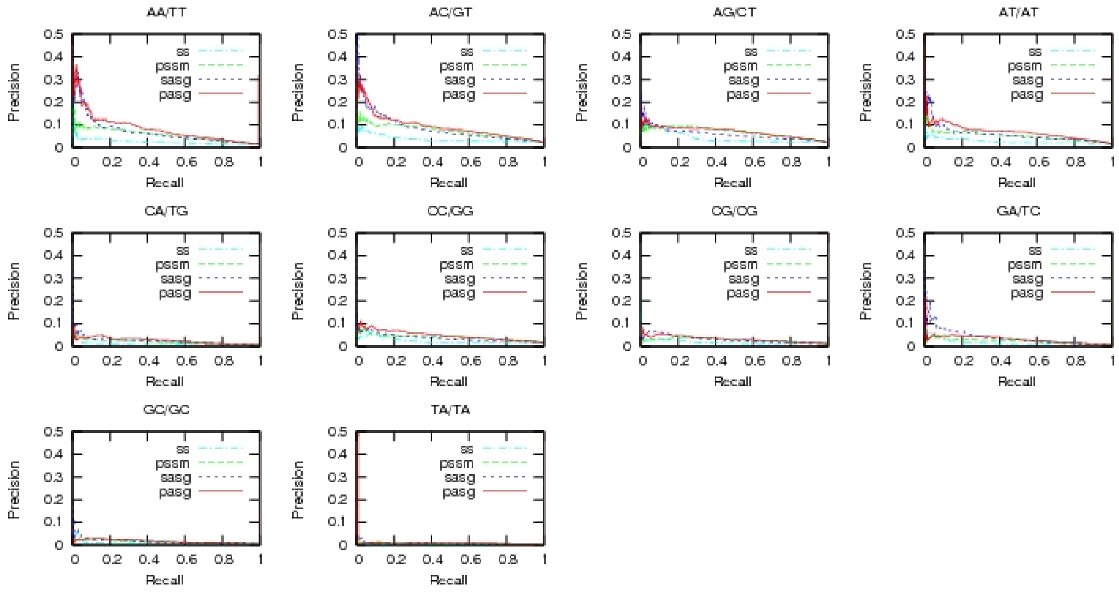

FigureS6: Dinucleotide step contact prediction in various secondary structures.

(a)

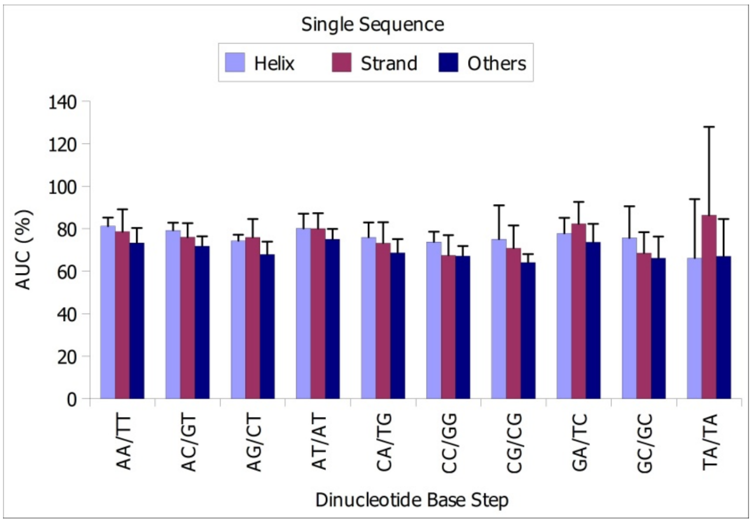

(b)

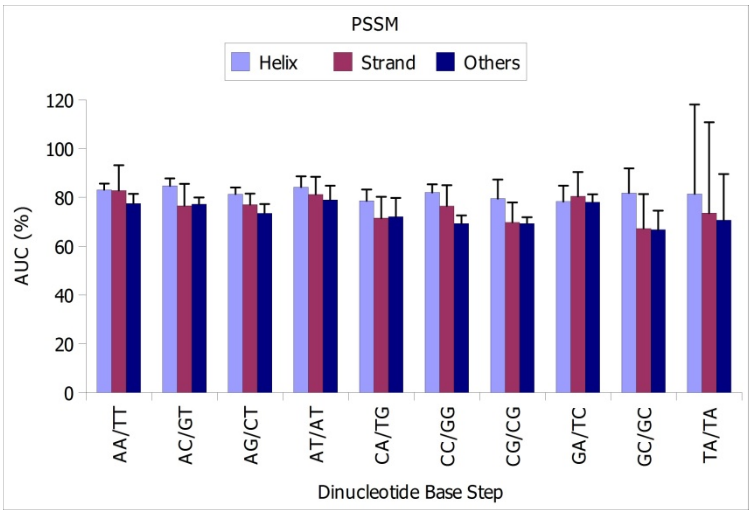

Figure S7: Dinucleotide step contact prediction in exposed and buried residues

(a)

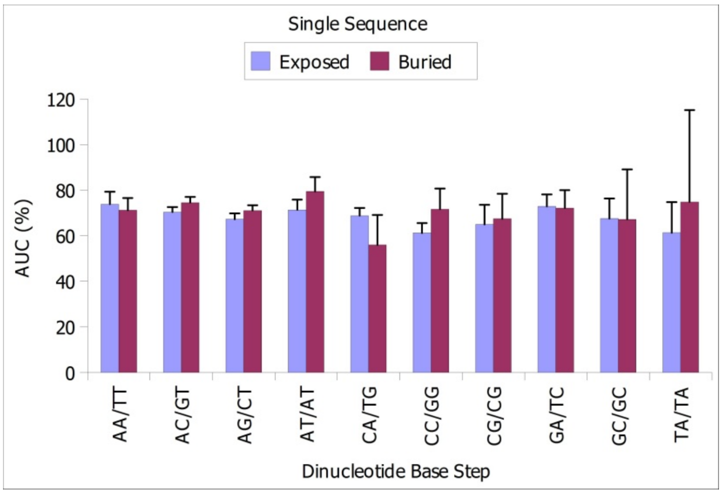

(b)

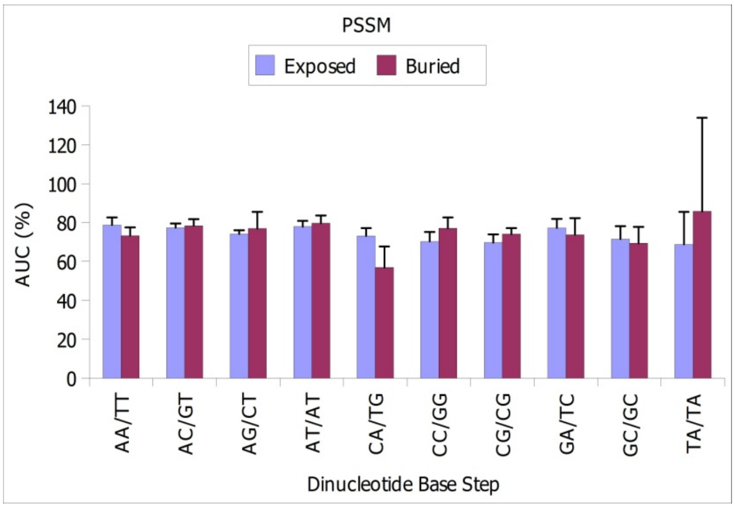

Supplement: Additional file 3 — Additional results of propensity and predictions in graphical format. [file 1472-6807-9-30-S3.pdf]
